# Longitudinal Multiparametric Quantitative MRI Evaluation of Graft Maturity Following Anterior Cruciate Ligament Reconstruction: A One-Year Prospective Observational Study

**DOI:** 10.3390/diagnostics16081121

**Published:** 2026-04-08

**Authors:** Jun-Jie Yang, Chao Ju, Long-Tao Yang, Ye-Xin Li, Mao-Sheng Wang, Jun-Jiao Hu, Jun Liu

**Affiliations:** 1 Department of Radiology, The Second Affiliated Hospital of Xinjiang Medical University, Ürümqi 830063, China; 2 Department of Radiology, The Second Xiangya Hospital, Central South University, Changsha 410011, China; 3 Department of Medical Imaging, Henan Provincial People’s Hospital & The People’s Hospital of Zhengzhou University, Zhengzhou 450003, China; 4 Department of Orthopaedics, The Second Xiangya Hospital, Central South University, Changsha 410011, China; 5 The Key Laboratory of Biomedical Information Engineering of Ministry of Education, Institute of Health and Rehabilitation Science, School of Life Science and Technology, Xi’an Jiaotong University, Xi’an 710049, China; 6 Clinical Research Center for Medical Imaging in Hunan Province, Department of Radiology Quality Control Center in Hunan Province, Changsha 410011, China

**Keywords:** anterior cruciate ligament, anterior cruciate ligament reconstruction, quantitative MRI, graft maturation, ligamentization, return to sport, patient-reported outcomes

## Abstract

**Background/Objectives:** Objective, non-invasive biomarkers are needed to track anterior cruciate ligament (ACL) graft maturation and support individualized return-to-sport decisions. This study evaluated a single-session multiparametric quantitative MRI (qMRI) protocol for longitudinal assessment of ACL graft microstructural evolution and its association with patient-reported outcomes. **Methods:** Twenty-eight patients undergoing primary ACL reconstruction with hamstring autografts underwent multiparametric qMRI (T1, T2*, R2*, and PD mapping) at 1, 3, 6, and 12 months. The contralateral native ACL served as a within-subject control. IKDC, Lysholm, and VAS scores were recorded at each visit. Linear mixed-effects models were used to test longitudinal changes. Correlations of baseline-normalized changes between adjacent visits were used to evaluate imaging–clinical associations. **Results:** All qMRI parameters changed significantly over time (all *p* < 0.001). At 1 month, T1, PD, and T2* were lower and R2* higher than the contralateral native ACL (all *p* < 0.001). Thereafter, T1, PD, and T2* increased and R2* decreased, with most metrics approaching contralateral values by 3–6 months (all *p* < 0.05), and changes entered a plateau after 6 months (all *p* > 0.05). IKDC, Lysholm, and VAS improved over time (all *p* < 0.001), mainly before 6 months. Greater early T2* increases and R2* decreases (1–3 months) were associated with less pain relief and smaller Lysholm improvement (*p* < 0.05); no significant associations were observed from 6–12 months. **Conclusions:** Single-session multiparametric qMRI sensitively captures ACL graft maturation and highlights 3–6 months as a critical remodeling window, providing objective biomarkers to complement clinical assessment for individualized rehabilitation monitoring and return-to-sport timing.

## 1. Introduction

With increasing participation in competitive sports and escalating training intensities, anterior cruciate ligament (ACL) injury has become an increasingly prevalent challenge in sports medicine [1,2]. In the United States alone, more than 120,000 cases occur annually, imposing a substantial clinical burden [3]. ACL reconstruction (ACLR) remains the gold standard for restoring knee stability following ACL rupture [4,5,6]. However, despite continual advances in surgical techniques, postoperative outcomes vary markedly across patients, influenced by surgical approach, graft choice, and rehabilitation protocols [7]. Therefore, determining the appropriate timing for return to sport (RTS) remains challenging. Returning too early may increase the risk of secondary injury, whereas delaying RTS may compromise functional recovery and athletic performance, particularly in young, high-level athletes [8]. In this context, reliable methods for assessing graft recovery are essential to optimize RTS timing.

Currently, RTS evaluation in clinical practice primarily relies on functional performance testing and patient-reported outcome measures [9,10]. However, RTS criteria remain heterogeneous, with no universally accepted consensus, and the effectiveness of RTS test batteries in reducing the risk of graft re-rupture and contralateral ACL injury is yet to be fully established [7,9]. Histopathological examination can directly reveal graft tissue changes but is invasive and unsuitable for longitudinal follow-up. Hence, non-invasive, objective, and accurate imaging-based assessment methods are of critical importance.

Conventional imaging modalities, such as standard morphological magnetic resonance imaging (MRI) and ultrasound, lack the sensitivity to adequately characterize the microstructural and biochemical properties of the graft [11,12]. Furthermore, recent studies suggest that indirect MRI findings, such as ATT, FTR, and the coronal LCL sign, may reflect residual knee instability after ACL reconstruction, but their associations with postoperative function remain limited [13,14]. This highlights the need for objective imaging biomarkers that can better characterize graft maturation and complement clinical assessment. Quantitative MRI (qMRI) overcomes these limitations by providing objective metrics that reflect the biological process of graft ligamentization [11]. However, relying on a single quantitative parameter may offer an incomplete evaluation. To address the challenge, we employed the MULTIPLEX technique [15]. Utilizing a dual flip-angle, multi-echo GRE sequence, this protocol enables the simultaneous, single-scan acquisition of high-resolution, B1-corrected T1, T2*/R2*, and PD maps with high SNR efficiency.

Leveraging this comprehensive framework, the present study aimed to quantitatively evaluate the longitudinal changes in graft healing across multiple dimensions without increasing scan times. We hypothesized that these multiparametric biomarkers would reliably reflect the temporal evolution of graft microstructure, thereby providing an objective basis for safe RTS decision-making and potentially mitigating the risk of secondary ACL injury.

## 2. Materials and Methods

### 2.1. Study Design and Participants

This single-center, longitudinal observational study consecutively enrolled 28 patients who underwent primary anterior cruciate ligament reconstruction (ACLR) at the Department of Sports Medicine, the Second Xiangya Hospital of Central South University, between January and December 2022. All participants underwent quantitative MRI at 1 (V1) and at 3 (V2), 6 (V3), and 12 months (V4). Inclusion criteria were primary ACLR, autologous hamstring graft (gracilis and semitendinosus), no known central or peripheral nervous system disease, and absence of cardiopulmonary or metabolic disorders; exclusion criteria comprised prior knee or lower-limb surgery, any ligamentous laxity not attributable to ACL injury, signs or symptoms of rheumatoid arthritis, autoimmune disease, or diabetes, inability or unwillingness to undergo postoperative MRI follow-up, and multi-ligament rupture or injury. A standardized case database was established to capture demographic information, contact details, quantitative MRI data, date and diagnosis at initial presentation, healing status of the injured site, and diagnoses at each follow-up; written informed consent was obtained from all participants prior to enrollment. At the first follow-up (V1, 1 month postoperatively), images of the contralateral anterior cruciate ligament were acquired as a within-subject healthy control.

### 2.2. Surgical Procedure and Postoperative Rehabilitation

All patients underwent the same arthroscopic single-bundle anterior cruciate ligament reconstruction under general anesthesia, performed by three sports medicine surgeons, each with at least 15 years of experience in knee surgery. With the patient in the supine position, standard skin preparation and draping were performed. Diagnostic arthroscopy was first carried out through the anteromedial and anterolateral portals to evaluate the extent of anterior cruciate ligament injury and to assess for concomitant meniscal or articular cartilage lesions. The graft was prepared using the semitendinosus and gracilis tendons. Through an approximately 3 cm longitudinal incision medial to the tibial tubercle, the tendons were harvested with a tendon stripper. Both ends of the tendons were whipstitched and then folded to prepare the graft, with a final graft diameter of 8 mm. After graft preparation, bone tunnel creation was performed under arthroscopic visualization [16]. The femoral tunnel was created independently through the anteromedial portal, with its position placed near the anatomic femoral footprint of the ACL remnant, close to the anteromedial bundle insertion site, in order to satisfy the requirements of anatomic reconstruction. The tibial tunnel was created in an outside-in manner, drilled from the anteromedial tibial cortex toward the center of the native ACL tibial footprint. The graft was then passed through the tibial tunnel and advanced into the femoral tunnel. On the femoral side, fixation was achieved using a cortical suspensory fixation device (Endobutton; Smith & Nephew, Memphis, TN, USA). The knee was then repeatedly flexed and extended several times to adequately pretension the graft while checking for any graft impingement or entrapment throughout the full range of motion. After confirming the absence of impingement, tibial fixation was performed with an interference screw (DePuy Mitek, Raynham, MA, USA) at approximately 30° of knee flexion while maintaining a posterior drawer force. Following fixation, intraoperative assessment of range of motion and stability was repeated to confirm satisfactory graft position, secure fixation, absence of significant impingement, and restoration of knee stability. The joint cavity and incision were then thoroughly irrigated, the wound was closed in layers, and the patient returned to the ward with a knee brace in place.

Postoperative rehabilitation followed a standardized protocol. Patients wore a knee brace for 6–8 weeks and ambulated with crutches while avoiding strenuous activity and further trauma. Ankle pump exercises were performed 300 times daily. Muscle strengthening consisted primarily of straight-leg-raise exercises, performed 300–500 times per day. Knee flexion exercises were initiated during the first postoperative week, with flexion training performed 1–2 times daily and a target flexion angle of 90°. The brace was adjusted progressively to allow increased flexion over time: 30° at discharge, 45° at postoperative week 2, 75° at postoperative week 3, and 90° at postoperative week 4.

### 2.3. Clinical Outcome Assessment

Clinical outcomes were evaluated at each follow-up (1, 3, 6, and 12 months postoperatively) using a standardized battery of patient-reported outcome measures (PROMs). The International Knee Documentation Committee (IKDC) Subjective Knee Form was used to assess overall knee function, symptoms, and sports activity levels. The Lysholm Knee Scoring Scale was employed to evaluate knee-specific symptoms, with a focus on instability, locking, and swelling. Additionally, knee pain intensity was quantified using the Visual Analog Scale (VAS), ranging from 0 (no pain) to 10 (worst imaginable pain). All clinical assessments were conducted by an independent sports medicine physician who was blinded to the MRI findings to minimize bias.

### 2.4. Imaging Procedure

All MRI examinations were performed at the Second Xiangya Hospital of Central South University using a 3.0 T scanner (uMR 790, United Imaging Healthcare, Shanghai, China). The imaging protocol utilized a 3D multi-parametric sequence (MULTIPLEX; United Imaging Healthcare, Shanghai, China). This technique features dual flip angles (FAs) and multi-echo acquisition, allowing for the simultaneous generation of both qualitative images and quantitative maps. In this study, the generated images included T1-weighted (T1W) and proton density-weighted (PDW) images, as well as T1, T2*, R2*, and PD maps. Image quality was visually inspected by experienced radiologists. The key parameters for the MULTIPLEX sequence were as follows: TR = 34.8 ms; echo times (TEs) = 3.11, 7.12, 9.82, 13.82, 16.53, 20.54, and 23.24 ms; field of view (FOV) = 190 × 190 mm^2^; acquisition voxel size = 0.93 × 0.79 × 2.0 mm^3^; slice thickness = 2.0 mm (64 slices); and dual flip angles of 4° and 16°. The total acquisition time was 8 min.

### 2.5. Image Analysis

**Figure 1:**
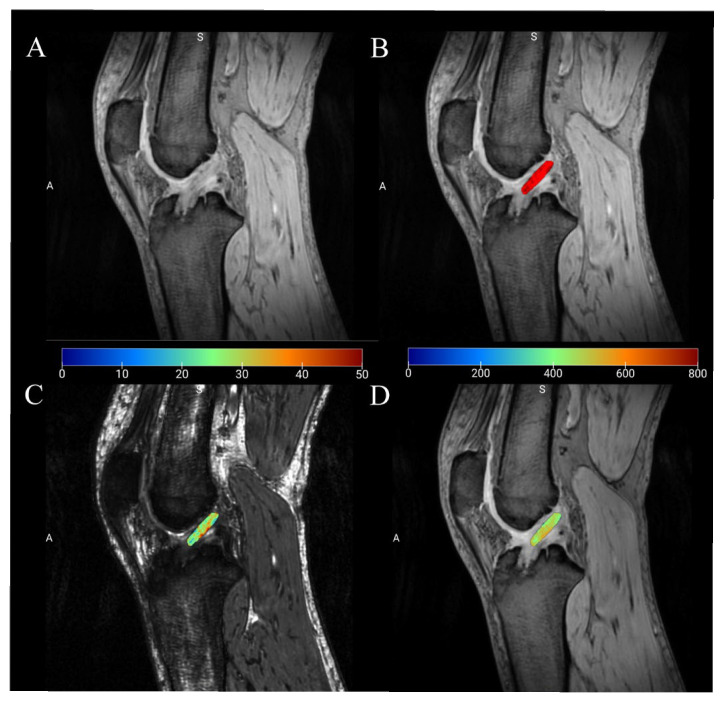
Schematic illustration of ROI delineation on quantitative parameter maps from MULTIPLEX multiparametric quantitative MRI. (**A**) PDWI image; (**B**) Delineation of the ROI on the PDWI; (**C**) Overlaying the ROI from the PDWI onto the T2* mapping; (**D**) Overlaying the ROI from the PDWI onto the PD mapping. The letters A and S represent anatomical orientation labels.

Image post-processing was performed using 3D Slicer software (version 5.8.1). Two radiologists, blinded to clinical information, manually delineated regions of interest (ROIs) covering the entire intra-articular portion of the ACL graft on the structural PD-weighted (PDW) images. To ensure accuracy, the femoral and tibial bone tunnels were strictly excluded to minimize susceptibility artifacts from fixation devices. The defined ROIs were subsequently utilized as masks and superimposed onto the corresponding spatially registered quantitative maps (including T1, T2*, R2*, and PD maps) to automatically extract the mean values for each parameter (Figure 1 and Figures S1 and S4).

### 2.6. Statistical Analysis

Continuous variables with normal distribution are presented as mean ± standard deviation, whereas categorical variables are expressed as number (percentage). Normality was assessed using the Shapiro–Wilk test. Longitudinal changes in quantitative MRI parameters and clinical scale scores were analyzed with linear mixed-effects models (LMMs), with time as a fixed effect and participant as a random effect. Post hoc pairwise comparisons were performed using estimated marginal means, and *p* values were adjusted with the false discovery rate (FDR) method. Correlation analyses were carried out between the relative changes in MRI parameters and scale scores across adjacent visits, normalized to baseline (V1) to eliminate individual baseline differences. To assess the reproducibility of the quantitative measurements, the intraclass correlation coefficient (ICC) was calculated based on the independent analysis of all images by two radiologists with 5 years of experience in musculoskeletal imaging. Specifically, intra-rater reliability was assessed by comparing the two repeated measurements performed by each radiologist, while inter-rater reliability was determined by comparing the mean values of the two measurements from the first radiologist against the mean values from the second radiologist. All statistical analyses were performed using R (version 4.3.3), while GraphPad Prism (version 10.4.1) was used exclusively for data visualization. Specifically, the irr package was used for reliability analysis (two-way random-effects model, absolute agreement), and the lme4, lmerTest, and car packages were used for linear mixed-effects models. Comparisons between the graft and the contralateral healthy ACL were conducted using the tidyverse package. The normality of paired differences was first assessed using the Shapiro–Wilk test. Based on the distribution, differences were analyzed using either the paired Student’s *t*-test (for normally distributed data) or the Wilcoxon signed-rank test (for non-normally distributed data). Pairwise comparisons were FDR-adjusted using the Benjamini–Hochberg method. ICC interpretation followed Koo and Li [17] (<0.50 poor, 0.50–0.75 moderate, 0.75–0.90 good, >0.90 excellent).

## 3. Results

### 3.1. Demographic Characteristics and Measurement Reliability

This study enrolled 28 participants, with a mean age of 23.29 ± 7.90 years. There were 19 males (67.9%) and 9 females (32.1%). The distribution of the affected side was balanced: 14 left (50.0%) and 14 right (50.0%). Overall, the sample represented a young, male-predominant cohort with equal left–right involvement (Table 1). qMRI measurements demonstrated high reproducibility, with inter-rater reliability showing good-to-excellent agreement (ICC > 0.82) and most intra-rater ICC values also indicating good-to-excellent agreement (ICC > 0.80). (Tables S1 and S2).

### 3.2. Longitudinal Changes in Quantitative MRI Parameters of the Intra-Articular ACL Graft and Comparison with Healthy Control

**Figure 2:**
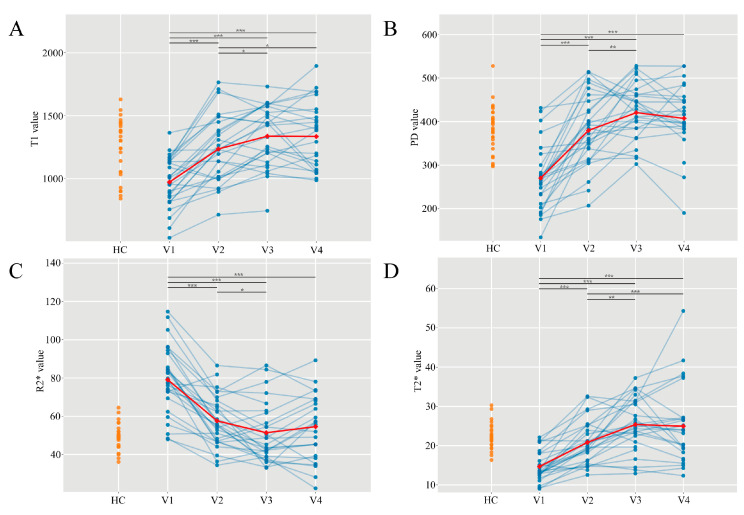
Longitudinal changes in quantitative MRI metrics after ACLR. (**A**–**D**) show T1, PD, R2*, and T2* value across four follow-up time points (V1–V4). Blue points and lines depict individual patient trajectories, and the red line shows the group-level trend given by the estimated marginal means from the mixed effects model. Orange points on the left represent healthy controls (HC). Horizontal brackets indicate pairwise comparisons between time points (*: *p* < 0.05, **: *p* < 0.01, ***: *p* < 0.001; FDR-adjusted).

**Figure 3:**
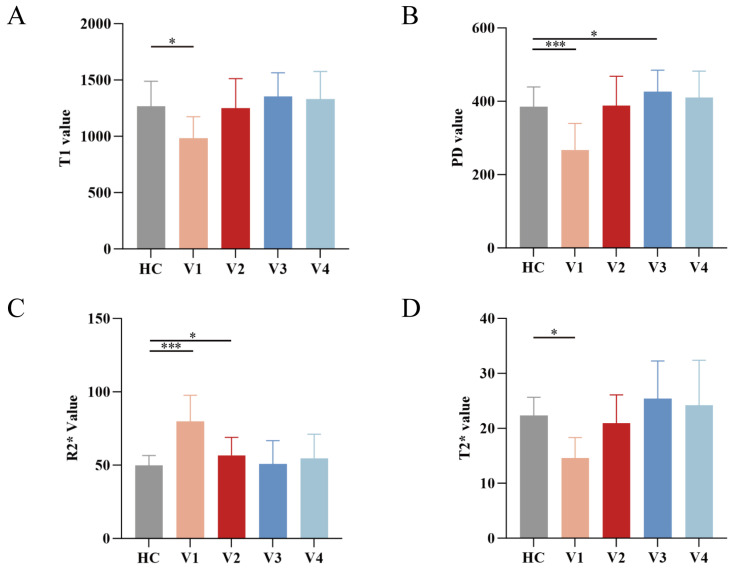
Pairwise comparisons of quantitative MRI metrics between contralateral native ACL and four follow-up visits. (**A**–**D**) show the comparisons of T1, PD, R2*, and T2* values at four follow-up time points with healthy controls. Horizontal brackets indicate pairwise comparisons between time points (*: *p* < 0.05, ***: *p* < 0.001; FDR-adjusted).

T1 values exhibited a significant main effect of time (F = 42.39, *p* < 0.001). Longitudinal pairwise contrasts revealed a marked increase from V1 to V2 (*p*_V1–V2_ < 0.001) and a further rise at V3 (*p*_V2–V3_ = 0.012), followed by stability between V3 and V4 (*p*_V3–V4_ = 0.95) (Figure 2A). Relative to healthy controls (HCs), T1 values at V1 were significantly lower (*p* < 0.001). However, no significant differences were observed from V2 onward (all *p* > 0.05) (Figure 3A). PD values similarly demonstrated a significant time effect (F = 47.88, *p* < 0.001), increasing continuously from V1 to V3 (*p*_V1–V2_ < 0.001, *p*_V2–V3_ = 0.007), with no significant change observed between V3 and V4 (*p*_V3–V4_ = 0.371) (Figure 2B). Compared with HCs, PD values were significantly lower at V1 (*p* < 0.001), exceeded control levels at 6 months (*p* = 0.016), and returned to non-significant levels by 12 months (*p* = 0.129); notably, no difference was found at V2 (*p* = 0.897) (Figure 3B). R2* values showed a significant time effect (F = 49.47, *p* < 0.001) but followed a declining trend from V1 to V3 (*p*_V1–V2_ < 0.001, *p*_V2–V3_ = 0.024), stabilizing between V3 and V4 (*p*_V3–V4_ = 0.249) (Figure 2C). In comparison with HCs, R2* values were significantly elevated at V1 (*p* < 0.001) and V2 (*p* = 0.04) but decreased to the level of HCs by V3 and V4 (*p* > 0.05) (Figure 3C). T2* values also presented a significant main effect of time (F = 30.53, *p* < 0.001), rising sharply from V1 to V2 (*p*_V1–V2_ < 0.001) and further increasing to V3 (*p*_V2–V3_ < 0.001), followed by a plateau from V3 to V4 (*p*_V3–V4_ = 0.742) (Figure 2D). Compared with HCs, T2* values were significantly lower at V1 (*p* < 0.001). These differences were resolved by 3 months (*p* = 0.339) and remained non-significant at 6 and 12 months (*p* > 0.05) (Figure 3D). Detailed estimated marginal means, pairwise comparisons, and comparisons with healthy controls are presented in Tables S3 and S4.

### 3.3. Longitudinal Changes in Clinical Scale Scores

**Figure 4:**
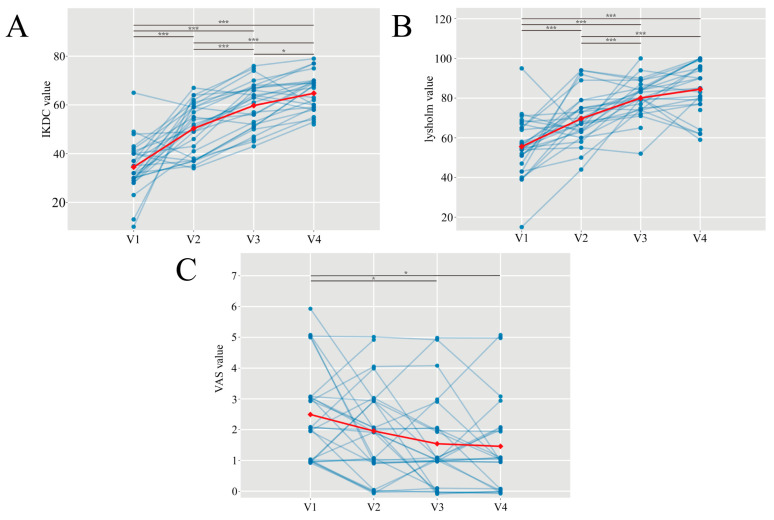
Longitudinal changes in clinical scale scores after ACLR. (**A**–**C**) show IKDC, Lysholm, and VAS scores across four follow-up visits (V1–V4). Blue points and lines depict individual trajectories, and the red line shows the group-level trend given by the estimated marginal means from the mixed-effects model. Horizontal brackets indicate pairwise comparisons between time points (*: *p* < 0.05, ***: *p* < 0.001; FDR-adjusted).

Figure 4 and Table S3 illustrate the longitudinal trajectories of clinical scores across four follow-up visits after ACL reconstruction. In a mixed-effects model, IKDC, Lysholm, and VAS scores all improved significantly over time (all *p* < 0.001). Pairwise contrasts showed that IKDC scores increased significantly from V1 to V2 (*p*_V1–V2_ < 0.001) and continued to rise through V3 (P_V2–V3_ < 0.001), with a further significant increase observed between V3 and V4 (P_V3–V4_ = 0.035). The Lysholm scores markedly improved from V1 to V2 (*p*_V1–V2_ < 0.001), with further gains from V2 to V3 (P_V2–V3_ < 0.001), and no significant change from V3 to V4 (P_V3–V4_ = 0.110). For pain relief assessed by VAS scores, although no significant differences were observed between adjacent time points (all *p* > 0.05), significant improvements relative to V1 were achieved at 6 months (*p*_V1–V3_ = 0.006) and remained significant at 12 months (P_V1–V4_ = 0.005).

### 3.4. Associations Between Quantitative MRI Metrics and Clinical Scale Scores

**Figure 5:**
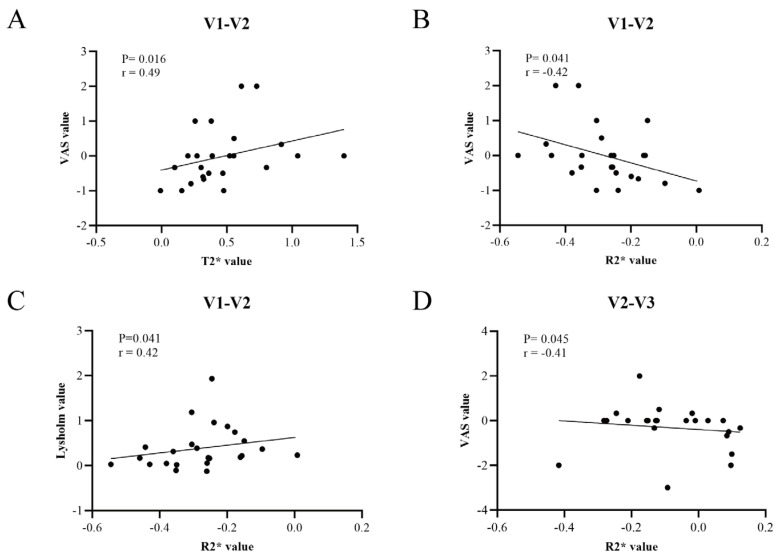
Correlation between quantitative MRI parameters and clinical outcomes after ACLR. (**A**–**D**) Correlations of MRI parameters and clinical scale changes across adjacent visits, expressed as relative change from V1. V1–V2: (V2 − V1)/V1; V2–V3: (V3 − V2)/V1.

In the correlation analysis of relative changes between adjacent follow-up visits, the change in T2* values from V1 to V2 was positively correlated with the change in VAS scores (r = 0.49, *p* = 0.016). Conversely, the change in R2* values during the same interval was negatively correlated with the change in VAS scores (r = −0.42, *p* = 0.041) and positively correlated with the improvement in Lysholm scores (r = 0.42, *p* = 0.041). Furthermore, from V2 to V3, the change in R2* values similarly exhibited a significant negative correlation with the change in VAS scores (r = −0.41, *p* = 0.045) (Figure 5).

## 4. Discussion

This study used a single-session multiparametric quantitative MRI (qMRI) protocol to longitudinally follow 28 patients for 12 months after ACL reconstruction (ACLR), with the aim of quantifying graft maturation and exploring imaging markers that may aid rehabilitation monitoring and return-to-sport (RTS) decision-making. Overall, qMRI metrics exhibited clear time-dependent trajectories, with the most pronounced remodeling changes occurring within the first 6 months before stabilizing. With the exception of PD, most parameters were no longer significantly different from the contralateral native ACL after 3 months. Clinically, IKDC, Lysholm, and VAS scores all improved steadily over the follow-up period. Moreover, in baseline-normalized correlation analyses, greater early increases in T2* and decreases in R2* from 1–3 months were associated with attenuated pain relief and smaller Lysholm improvements, whereas no significant imaging–clinical associations were observed from 6–12 months. Collectively, these imaging and clinical trends indicate a pivotal transition from the early healing phase to the remodeling phase between 3 and 6 months postoperatively. Multiparametric qMRI may therefore provide an objective complement to conventional assessments for tracking recovery and informing RTS decisions.

Direct histologic evidence of early ligamentization in humans remains limited, as ethical and safety concerns preclude routine early graft biopsies [18]. Drawing from preclinical animal models and available human literature, the ligamentization process is commonly categorized into three distinct stages [19,20]. These typically include an early healing phase spanning the first 6 months, a remodeling phase from approximately 6 to 12 months, and a final maturation phase commencing thereafter. In the immediate postoperative period, hamstring tendon autografts are presumed to retain their native “tendon-like” characteristics, featuring a more compact, organized collagen architecture and lower water content compared with the native ACL [21]. This biological profile aligns with our observation of lower T1, PD, and T2* values—and conversely higher R2* values—relative to the contralateral ACL at the initial time point. Between 1 and 6 months, biological processes such as inflammation, revascularization, and collagen matrix loosening likely occur [22,23], driving an increase in T1, PD, and T2* values (and a decrease in R2* values). From 6 to 12 months, as water content decreases, hypervascularity subsides, and collagen realigns [22], these quantitative metrics exhibited a trend toward normalization, reflecting ongoing tissue remodeling. The maturation phase is generally considered to begin beyond 12 months postoperatively, a period during which the graft progressively approaches a “native ACL–like” state. However, persistent ultrastructural disparities reportedly endure, most notably a shift in collagen fibril diameter from the bimodal pattern typical of a normal ACL to a predominantly unimodal distribution of smaller fibrils [24]. This is consistent with prior MRI studies demonstrating continued graft remodeling at 18–24 months postoperatively or beyond [21,25,26]. As our follow-up was limited to 12 months, this study could not fully capture the long-term imaging evolution corresponding to the final maturation phase. Accordingly, the close correspondence between these canonical histologic stages and the temporally ordered changes in T1, PD, T2*, and R2* observed in our cohort supports the feasibility of multiparametric qMRI as a noninvasive surrogate for tracking graft ligamentization and delineating clinically meaningful remodeling windows.

Historically, the assessment of graft maturation following ACL reconstruction (ACLR) has largely relied on conventional MRI sequences, with the signal-to-noise quotient (SNQ) serving as the predominant semi-quantitative metric. However, the reliability of SNQ is compromised by its susceptibility to confounding factors, including background noise variability, radiofrequency coil inhomogeneity, and inconsistencies in acquisition parameters across different scanners and protocols. Furthermore, SNQ primarily reflects nonspecific tissue water content (edema) and lacks the sensitivity required to quantify subtle microstructural alterations in collagen organization [12]. Consequently, there has been a paradigm shift toward qMRI techniques, which offer objective biomarkers capable of more accurately characterizing the intrinsic biophysical properties of the graft tissue.

The multi-parametric qMRI findings observed in this study align closely with prior longitudinal investigations utilizing UTE-T2*, T2*, and T1ρ mapping techniques [21,26,27,28]. Collectively, these studies demonstrate that autologous hamstring grafts exhibit marked elevations in T2, T1ρ, and associated relaxation metrics during the early postoperative period (approximately 1–6 months), reflecting underlying inflammation, revascularization, and collagen disorganization. These quantitative values typically peak or plateau around 3–6 months before gradually declining between 6 and 12 months, progressively approaching the baseline values of the contralateral native or healthy control ACL. Specifically, Chu et al., employing UTE-T2* mapping, reported that graft T2* values remained elevated throughout the first 6 months, followed by a significant decrease from 6 to 12 months [21]. Similarly, Yoshimizu et al. observed a pronounced reduction in the short-T2 component between 6 and 12 months, paralleling the T2* trajectory observed in our cohort [27]. Furthermore, Lansdown et al. demonstrated that both T1ρ (*p* = 0.042) and T2 (*p* = 0.036) values were significantly lower at 12 months compared with 6 months, supporting the concept that a critical phase of remodeling and maturation ensues after the 6-month mark [26]. Taken together, these studies substantiate the validity of our results and indicate that our multi-parametric qMRI protocol is highly sensitive to the pivotal “ligamentization” transition window occurring around 3–6 months post-ACLR. Accordingly, the expected transition of quantitative MRI parameters from the early healing phase to the remodeling phase at approximately 6 months postoperatively may serve as an early indicator of delayed graft maturation, potentially alerting clinicians to suboptimal recovery trajectories prior to overt changes in conventional clinical assessments. Building on these findings, the multi-parametric MRI protocol employed in this study offers distinct advantages for clinical translation. First, it allows for the efficient acquisition of multiple quantitative parameters within a clinically feasible timeframe. Second, the concordance and potential for cross-validation across different parameters enhance the overall robustness and diagnostic confidence of the findings. Crucially, the integration of T2*/R2* metrics—which capture collagen fiber ultrastructure—with PD/T1 metrics reflecting tissue hydration and vascularity provides a comprehensive and precise assessment of the graft maturation process.

Regarding the relationship between imaging biomarkers and clinical outcomes, previous investigations have predominantly relied on cross-sectional designs. However, such cross-sectional approaches may obscure inter-individual baseline heterogeneity [29,30,31]. In contrast, the present study employed a baseline-adjusted longitudinal analysis to more accurately capture dynamic associations between imaging changes and clinical trajectories. During the early postoperative phase (1–3 months), pronounced shifts in imaging metrics were significantly correlated with inferior clinical improvement. Specifically, a greater magnitude of decrease in R2* and increase in T2* values was associated with attenuated pain relief and diminished gains in Lysholm scores. This finding suggests that an early trend toward “normalization” relative to contralateral values may not necessarily indicate true histologic recovery. Rather, it likely reflects a pronounced inflammatory response and tissue edema. Furthermore, it may signal the collagen disorganization characteristic of active remodeling. Conversely, from 6 to 12 months, no significant associations were observed between changes in imaging metrics and improvements in clinical scores. This lack of correlation is consistent with previous reports [12,32,33,34]. A plausible explanation is that qMRI captures microstructural maturation, whereas clinical function depends more on macroscopic biomechanics. Another explanation is reduced score sensitivity at later stages. IKDC and Lysholm scores are subjective and may show ceiling effects [35,36,37]. Importantly, the absence of significant associations between quantitative MRI parameters and clinical scores during the 6–12 month remodeling phase is broadly consistent with recent evidence based on conventional MRI markers of residual knee instability. Genç et al. demonstrated that indirect MRI findings such as ATT distance, FTR angle, and the coronal LCL sign did not show consistent relationships with postoperative knee scores or functional hop test performance at 6 months after ACLR [13]. Similarly, Karatekin et al. observed that postoperative ATT and FTR abnormalities may persist after ACLR, but were not significantly associated with inferior clinical scores or functional deficits [14]. Taken together, these findings suggest that MRI-derived markers may more strongly reflect the biological or structural changes that occur during graft recovery, whereas their relationship with patient-reported outcomes or functional performance may not be entirely synchronous, particularly during the later remodeling phase. Overall, these findings highlight the limitations of single-domain evaluations. Consequently, a multimodal assessment framework is recommended. Objective qMRI biomarkers should be interpreted in conjunction with PROMs to provide a comprehensive assessment of recovery following ACLR.

This study acknowledges several limitations. First, the relatively small sample size (*n* = 28) and single-center design may restrict statistical power and the generalizability of the findings. Second, the 12-month follow-up period precludes the characterization of late-stage graft maturation (>12 months) and the evaluation of long-term clinical correlations. Third, despite the use of delta-based analyses to mitigate baseline heterogeneity, associations between imaging metrics and clinical function during the 6-to-12-month remodeling phase may have been attenuated by the inherent subjectivity and ceiling effects of standard PROMs (e.g., IKDC, Lysholm). Moreover, the absence of actual return-to-sport outcomes and reinjury data precludes the direct application of these qMRI findings as formal RTS decision criteria. Fourth, qMRI parameters, particularly T2* and R2*, remain susceptible to the magic angle effect; thus, despite standardized positioning, minor variations in knee flexion may have introduced measurement variability. Fifth, this study used a more conservative postoperative rehabilitation protocol than those commonly adopted in current practice, which may have influenced early graft loading and remodeling, thereby somewhat limiting the generalizability of the findings. Future research should adopt multicenter, prospective designs with larger cohorts and extended follow-up (≥24 months) to enhance external validity. To better capture late-phase recovery, we recommend supplementing PROMs with objective functional endpoints, such as isokinetic strength testing, hop tests, and biomechanical assessments. Additionally, minimizing systematic error from the magic angle effect will require rigorous positioning protocols or the adoption of acquisition strategies less sensitive to angular dependency. Finally, comparative MRI studies are warranted to investigate how different tunnel preparation techniques (such as all-inside versus anteromedial portal) influence graft maturation trajectories.

## 5. Conclusions

In conclusion, this study demonstrates the utility of a single-scan, multi-parametric qMRI protocol (incorporating T1, T2*, R2*, and PD mapping) for the comprehensive, longitudinal, and objective monitoring of graft maturation following ACL reconstruction. Our findings successfully characterized the microstructural evolution of the graft over the first 12 months, identifying the 3-to-6-month interval as a pivotal window for biological remodeling. These results underscore the potential of multi-parametric qMRI as a robust, non-invasive tool for assessing graft maturity, offering clinicians objective data to refine individualized rehabilitation strategies and return-to-sport decision-making.

## Figures and Tables

**Table 1:** Demographic Characteristics.

Characteristic	Value
Overall, *n*	28
Age, mean ± sd	23.29 ± 7.90
Sex	
Male, *n* (%)	19 (67.9%)
Female, *n* (%)	9 (32.1%)
Side of Injury ^α^	
Left, *n* (%)	14 (50.0%)
Right, *n* (%)	14 (50.0%)

^α^ Left and right indicate the laterality of the anterior cruciate ligament graft.

## Data Availability

The raw data supporting the conclusions of this article will be made available by the authors on request.

## Supplementary Materials

The following supporting information can be downloaded at: https://www.mdpi.com/article/10.3390/diagnostics16081121/s1, Table S1. Inter-rater reliability of quantitative MRI parameters at different follow-up time points. Table S2. Intra-rater reliability of quantitative MRI parameters assessed by two independent radiologists. Table S3. Longitudinal Changes in Quantitative MRI Parameters and Clinical Outcomes Following ACL Reconstruction. Table S4. Longitudinal Changes of Graft qMRI Parameters Compared to Healthy Reference. Figure S1. Representative pseudo-color T1 mapping images of the anterior cruciate ligament. Figure S2. Representative pseudo-color Proton Density (PD) mapping images of the anterior cruciate ligament. Figure S3. Representative pseudo-color R2* mapping images of the anterior cruciate ligament. Figure S4. Representative pseudo-color T2* mapping images of the anterior cruciate ligament.

## Abbreviations

The following abbreviations are used in this manuscript:

ACL	Anterior Cruciate Ligament
ACLR	Anterior Cruciate Ligament Reconstruction
FAs	Flip Angles
FDR	False Discovery Rate
FOV	Field of View
HC	Healthy Control
ICC	Intraclass Correlation Coefficient
IKDC	International Knee Documentation Committee
LMMs	Linear Mixed-Effects Models
MRI	Magnetic Resonance Imaging
PD	Proton Density
PDW	Proton Density-Weighted
PROMs	Patient-Reported Outcome Measures
qMRI	Quantitative Magnetic Resonance Imaging
ROI	Regions of Interest
RTS	Return to Sport
SNQ	Signal-to-Noise Quotient
T1W	T1-Weighted
TEs	Echo Times
TR	Repetition Time
UTE	Ultrashort Echo Time
VAS	Visual Analog Scale
