# Supplementary material for: Longitudinal Multiparametric Quantitative MRI Evaluation of Graft Maturity Following Anterior Cruciate Ligament Reconstruction: A One-Year Prospective Observational Study
Source: Diagnostics (Basel). 2026 Apr 8;16(8):1121. doi: 10.3390/diagnostics16081121 (PMC13114530; doi:10.3390/diagnostics16081121)
Supplement: Supplementary file 1 [file diagnostics-16-01121-s001.zip › diagnostics-4196955-supplementary.pdf]

Table S1. Inter-rater reliability of quantitative MRI parameters at different follow-up time points.

Sequence	Time Point (Months)	ICC (95%CL)	P value
T1	1	0.94 (0.88–0.97)	4.54E-15
	3	0.98 (0.96–0.99)	1.13E-21
	6	0.94 (0.86–0.97)	3.15E-13
	12	0.98 (0.95–0.99)	2.05E-18
$T2^*$	1	0.88 (0.76–0.94)	2.24E-10
	3	0.83 (0.67–0.92)	7.31E-09
	6	0.94 (0.87–0.97)	3.06E-14
	12	0.92 (0.83–0.97)	1.35E-11
PD	1	0.92 (0.83–0.96)	7.66E-13
	3	0.98 (0.96–0.99)	1.53E-21
	6	0.93 (0.85–0.97)	5.61E-13
	12	0.93 (0.86–0.97)	1.62E-12
$R2^*$	1	0.87 (0.74–0.94)	1.22E-09
	3	0.93 (0.85–0.97)	1.53E-13
	6	0.82 (0.64–0.91)	6.16E-08
	12	0.84 (0.68–0.93)	2.87E-08

Abbreviations: ICC, intraclass correlation coefficient; CI, confidence interval; T1, T1 mapping; T2*, T2* mapping; PD, PD mapping, $R2^*$, $R2^*$ mapping. ICC values indicate the degree of agreement: < 0.50, poor; 0.50–0.75, moderate; 0.75–0.90, good; > 0.90, excellent.

Table S2. Intra-rater reliability of quantitative MRI parameters assessed by two independent radiologists.

Sequence	rater	Time Point (Months)	ICC (95%CI)	P value
T1	1	1	0.90 (0.80–0.95)	1.81E-11
	1	3	0.98 (0.96–0.99)	5.84E-22
	1	6	0.90 (0.75–0.95)	4.16E-08
	1	12	0.96 (0.92–0.98)	5.76E-16
	2	1	0.96 (0.93–0.98)	1.32E-17
	2	3	0.98 (0.96–0.99)	1.54E-21
	2	6	0.96 (0.91–0.98)	1.54E-16
	2	12	0.98 (0.95–0.99)	5.33E-17
$T2^*$	1	1	0.74 (0.52–0.87)	1.83E-06
	1	3	0.91 (0.81–0.96)	2.03E-12
	1	6	0.91 (0.81–0.96)	4.51E-10
	1	12	0.91 (0.76–0.97)	3.29E-07
	2	1	0.81 (0.64–0.91)	4.66E-08
	2	3	0.82 (0.64–0.91)	2.52E-08
	2	6	0.90 (0.77–0.95)	8.63E-10
	2	12	0.96 (0.92–0.98)	1.87E-15
PD	1	1	0.85 (0.71–0.93)	1.09E-09
	1	3	0.92 (0.84–0.96)	9.83E-13
	1	6	0.93 (0.85–0.97)	2.49E-13
	1	12	0.85 (0.69–0.93)	4.28E-08
	2	1	0.96 (0.91–0.98)	5.95E-16
	2	3	0.97 (0.93–0.98)	6.72E-18
	2	6	0.93 (0.84–0.97)	2.46E-11
	2	12	0.93 (0.84–0.97)	2.04E-12
$R2^*$	1	1	0.70 (0.45–0.85)	1.35E-05
	1	3	0.83 (0.66–0.92)	9.54E-09
	1	6	0.72 (0.47–0.86)	8.58E-06
	1	12	0.62 (0.24–0.82)	0.0014195
	2	1	0.91 (0.81–0.96)	6.41E-12
	2	3	0.92 (0.84–0.96)	1.89E-13
	2	6	0.92 (0.83–0.96)	1.86E-12
	2	12	0.91 (0.81–0.96)	5.11E-11

Abbreviations: ICC, intraclass correlation coefficient; CI, confidence interval; T1, T1 mapping; T2*, T2* mapping; PD, PD mapping, $R2^*$, $R2^*$ mapping. ICC values indicate the degree of agreement: < 0.50, poor; 0.50–0.75, moderate; 0.75–0.90, good; > 0.90, excellent.

Table S3. Longitudinal Changes in Quantitative MRI Parameters and Clinical Outcomes Following ACL Reconstruction.

Variables	V1 (1 mo)		V2 (3 mo)		V3 (6 mo)		V4 (12 mo)		$P_{overall}$	P_{V1-V2}	P_{V1-V3}	P_{V1-V4}	P_{V2-V3}	P_{V2-V4}	P_{V3-V4}
	emmean	SE	emmean	SE	emmean	SE	emmean	SE							
**qMRI Parameters**															
T1	973	46.7	1236	46.7	1337	47	1335	47.8	< 0.001	< 0.001	< 0.001	< 0.001	0.012	0.014	0.948
PD	270	14.3	380	14.3	420	14.5	407	14.8	< 0.001	< 0.001	< 0.001	< 0.001	0.007	0.068	0.371
R2*	79.1	3.07	57.6	3.07	51.4	3.1	54.6	3.15	< 0.001	< 0.001	< 0.001	< 0.001	0.024	0.249	0.249
T2*	14.7	1.3	20.8	1.3	25.4	1.31	25	1.35	< 0.001	< 0.001	< 0.001	< 0.001	< 0.001	0.003	0.742
**Clinical Outcomes**															
IKDC	34.5	1.81	50.4	1.81	59.7	1.84	64.8	1.87	< 0.001	< 0.001	< 0.001	< 0.001	< 0.001	< 0.001	0.035
Lysholm	55.6	2.2	69.7	2.2	80.1	2.23	84.5	2.27	< 0.001	< 0.001	< 0.001	< 0.001	< 0.001	< 0.001	0.110
VAS	2.49	0.277	1.96	0.277	1.54	0.281	1.46	0.285	0.003	0.139	0.006	0.005	0.196	0.149	0.784

Values are presented as estimated marginal means (emmeans) and standard errors (SE) derived from linear mixed-effects models.

Abbreviations: IKDC, International Knee Documentation Committee Subjective Knee Form; VAS, Visual Analog Scale; V1, 1 month postoperatively; V2, 3 months; V3, 6 months; V4, 12 months. P values indicate the significance of the fixed effect of time (overall) and post-hoc pairwise comparisons (P values are FDR-adjusted using the Benjamini–Hochberg method).

Table S4. Longitudinal Changes of Graft qMRI Parameters Compared to Healthy Reference.

MRI Parameter	Time Point	Graft Values	P Value
T1		Healthy Reference :1265.37 ± 223.30	—
	1 Month	983.80 ± 189.65	**< 0.001**
	3 Months	1250.58 ± 261.38	0.860
	6 Months	1354.78 ± 209.28	0.256
	12 Months	1329.46 ± 246.67	0.466
PD		Healthy Reference: 385.36 ± 53.91[a]	—
	1 Month	267.49 ± 72.44	**< 0.001**
	3 Months	388.28 ± 80.01	0.897
	6 Months†	428.34 [392.16,460.32]	**0.016**
	12 Months	409.82 ± 72.40	0.129
R2*		Healthy Reference: 49.76 ± 6.74	—
	1 Month	79.84 ± 17.87	**< 0.001**
	3 Months	56.66 ± 12.25	**0.040**
	6 Months	50.86 ± 15.84	0.770
	12 Months	54.73 ± 16.32	0.233
T2 *		Healthy Reference: 22.30 ± 3.35[b]	—
	1 Month	14.60 ± 3.71	**< 0.001**
	3 Months	20.95 ± 5.12	0.339
	6 Months	25.43 ± 6.80	0.115
	12 Months†	23.88 [18.79,26.94]	0.595

Note: Data are presented as mean ± SD unless otherwise indicated.

†Data presented as median [IQR] and analyzed using the Wilcoxon signed-rank test due to non-normal distribution. For PD at 6 months, the corresponding healthy reference median was 393.74 [353.75, 415.19]. For T2* at 12 months, the corresponding healthy reference median was 21.66 [20.76, 23.37]. P values are FDR-adjusted using the Benjamini–Hochberg method.

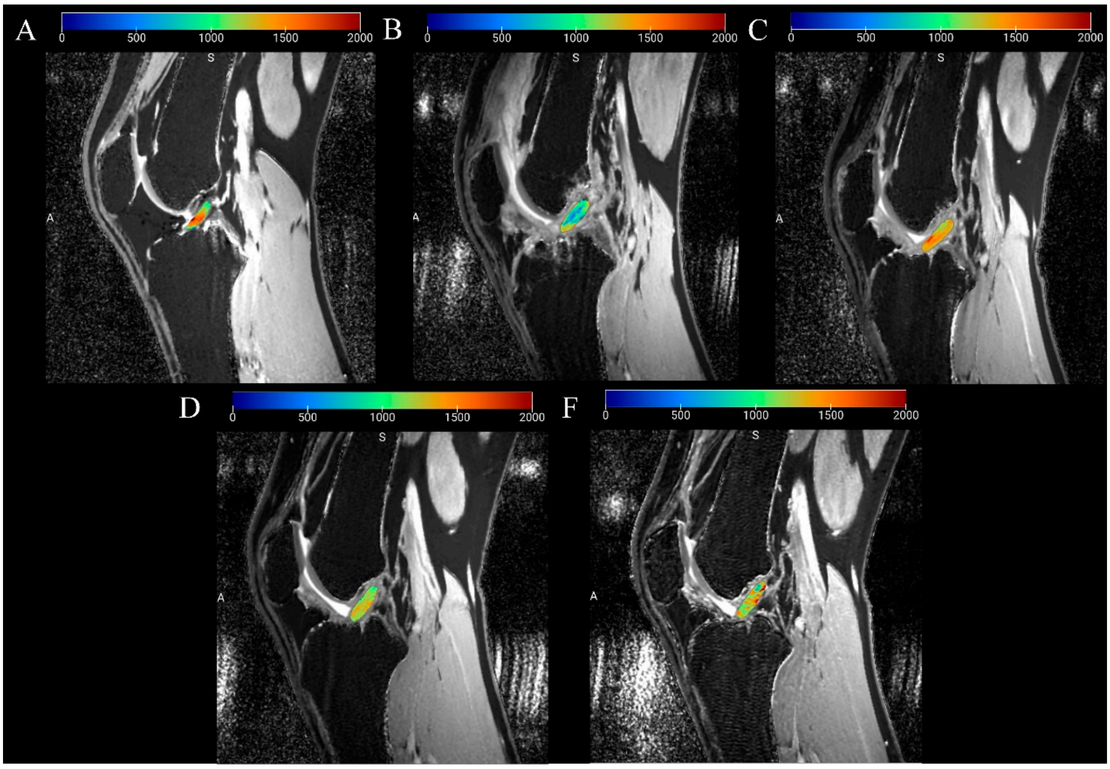

Figure S1. Representative pseudo-color T1 mapping images of the anterior cruciate ligament. (A) The intact native ACL of the contralateral healthy knee serves as the control. (B–F) Longitudinal changes in the ACL graft at different follow-up time points: (B) 1 month, (C) 3 months, (D) 6 months, and (F) 12 months postoperatively. The color scale represents the T1 relaxation time. Warmer colors (red/orange) indicate higher T1 values, while cooler colors (blue/green) indicate lower T1 values.

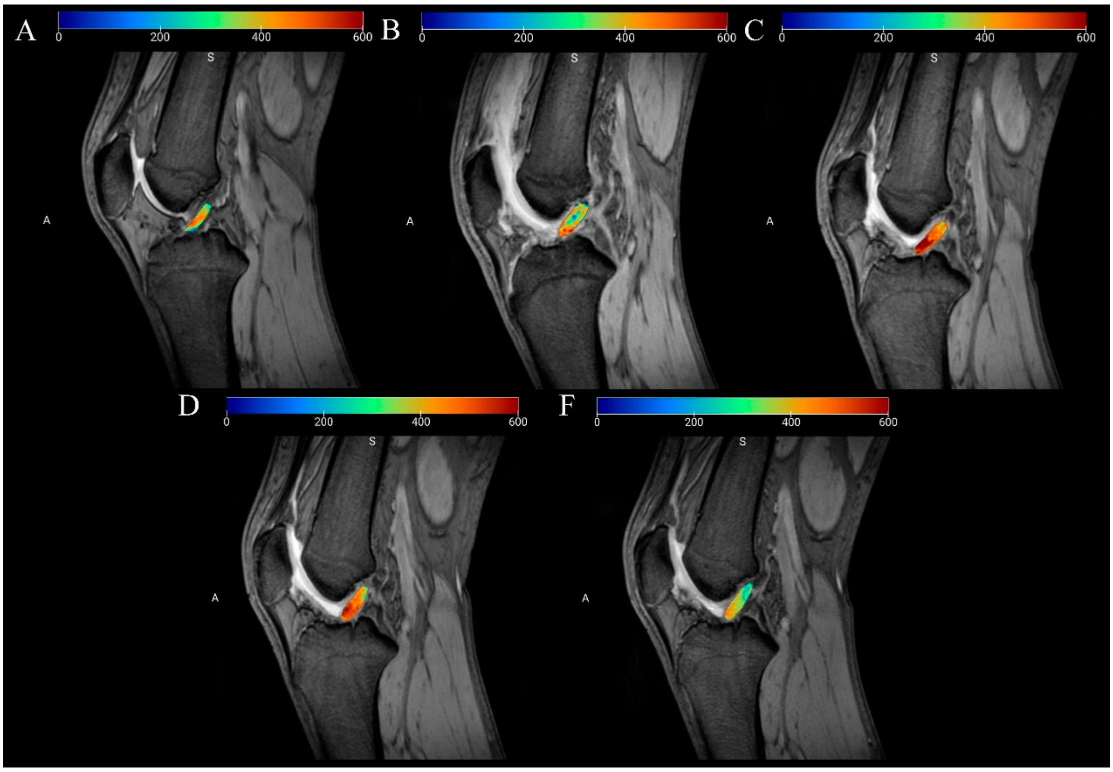

Figure S2. Representative pseudo-color Proton Density (PD) mapping images of the anterior cruciate ligament. (A) The intact native ACL of the contralateral healthy knee serves as the control. (B–F) Longitudinal changes in the ACL graft at different follow-up time points: (B) 1 month, (C) 3 months, (D) 6 months, and (F) 12 months postoperatively. The color scale represents the PD values. Warmer colors (red/orange) indicate higher proton density, while cooler colors (blue/green) indicate lower proton density.

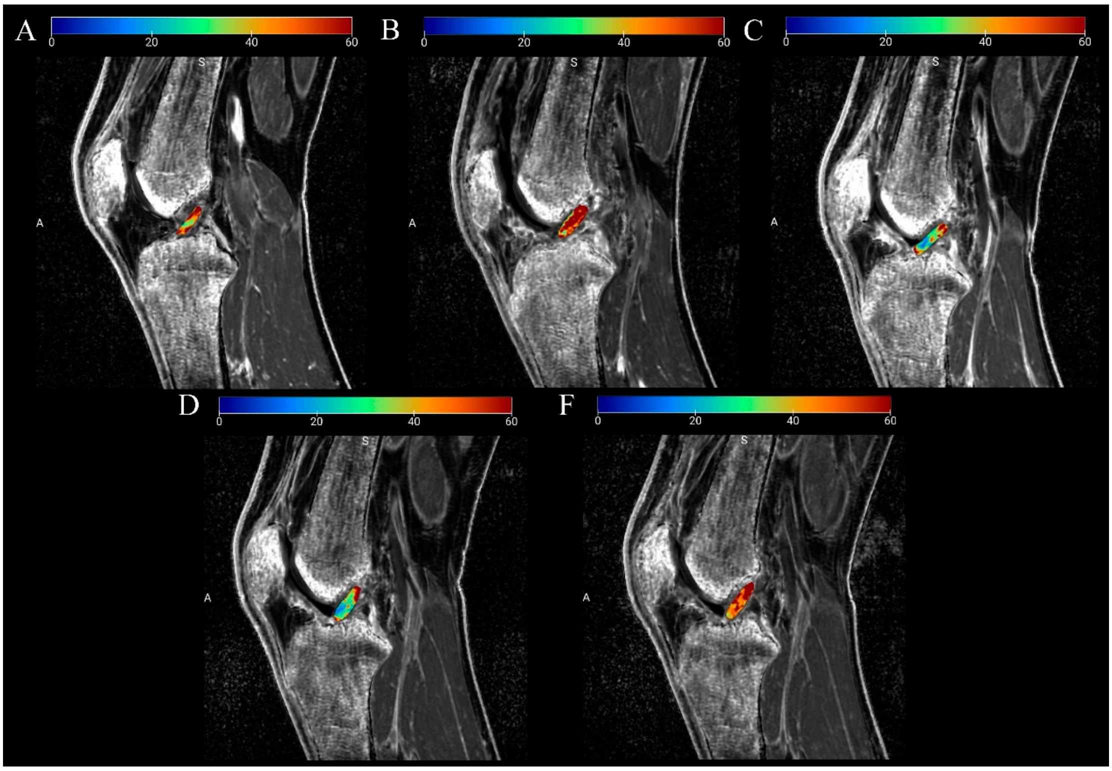

Figure S3. Representative pseudo-color R2* mapping images of the anterior cruciate ligament. (A) The intact native ACL of the contralateral healthy knee serves as the control. **(B–F)** Longitudinal changes in the ACL graft at different follow-up time points: **(B)** 1 month, **(C)** 3 months, **(D)** 6 months, and **(F)** 12 months postoperatively. The color scale represents the R2* values. Warmer colors (red/orange) indicate higher R2* values, while cooler colors (blue/green) indicate lower R2* values.

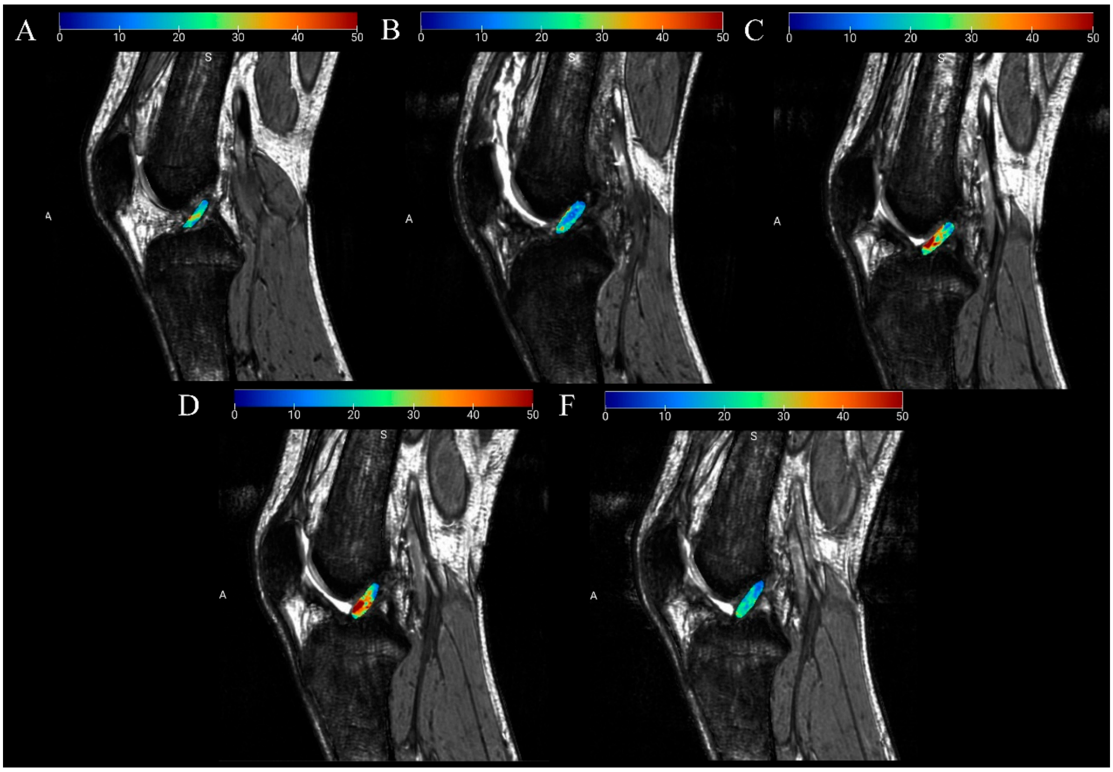

Figure S4. Representative pseudo-color T2* mapping images of the anterior cruciate ligament. (A) The intact native ACL of the contralateral healthy knee serves as the control. **(B–F)** Longitudinal changes in the ACL graft at different follow-up time points: **(B)** 1 month, **(C)** 3 months, **(D)** 6 months, and **(F)** 12 months postoperatively. The color scale represents the T2* values. Warmer colors (red/orange) indicate higher T2* values, while cooler colors (blue/green) indicate lower T2* values.
